# FLASH Radiotherapy Enhances the Therapeutic Ratio in an Embryonic In Vivo Model of Pancreatic Carcinoma

**DOI:** 10.1021/acsami.5c19638

**Published:** 2025-11-07

**Authors:** Noemi Giannini, Alessandra Gonnelli, Giovanni Gadducci, Paola Puccini, Andrea Cavalieri, Luigi Masturzo, Jake Harold Pensavalle, Mariagrazia Celentano, Fabio Di Martino, Federico Di Cocco, Cristian Scatena, Antonio Giuseppe Naccarato, Michele Menicagli, Patrizia Sarogni, Valentina Frusca, Dania Cioni, Giovanni Donato Aquaro, Valerio Voliani, Fabiola Paiar

**Affiliations:** † Center for Nanotechnology Innovation@NEST−Istituto Italiano di Tecnologia, Piazza San Silvestro 12, 56127 Pisa, Italy; ‡ Radiation Oncology Unit, Pisa University Hospital “Azienda Ospedaliero-Universitaria Pisana”, Via Roma 67, Pisa 56126, Italy; § Department of Translational Research and New Technologies in Medicine and Surgery, University of Pisa, Pisa 56126, Italy; ∥ Centro Pisano Multidisciplinare Sulla Ricerca e Implementazione Clinica Della Flash Radiotherapy (CPFR), University of Pisa, Pisa 56126, Italy; ⊥ Center for Instrument Sharing of the University of Pisa (CISUP), University of Pisa, Pisa 56126, Italy; # Sordina IORT Technologies S.p.A., Research and development, Aprilia 04011, Italy; ∇ Unit of Medical Physics, Pisa University Hospital “Azienda Ospedaliero-Universitaria Pisana”, via Roma 67, Pisa 56126, Italy; ○ Division of Pathology, Department of Translational Research and New Technologies in Medicine and Surgery, University of Pisa, Pisa 56126, Italy; ◆ Department of Oncology, 9257 Pisa University Hospital, Pisa 56100, Italy; ¶ Fondazione Pisana per la Scienza ONLUS, via Ferruccio Giovannini 13, S. Giuliano Terme, 56017 Pisa, Italy; †† Scuola Superiore Sant’Anna, Piazza Martiri della Libertà, 33, Pisa 56127, Italy; ‡‡ Unit of Radiology, Pisa University Hospital “Azienda Ospedaliero-Universitaria Pisana”, via Roma 67, Pisa 56126, Italy; §§ Department of Pharmacy, School of Medical and Pharmaceutical Sciences, University of Genoa, Viale Cembrano 4, Genoa 16148, Italy; ∥∥ Centro 3R- Centro Interuniversitario per la promozione dei principi delle 3R nella didattica e nella ricerca, 10129 Turin, Italy

**Keywords:** FLASH, radiotherapy, alternative biomodels, chorioallantoic membrane model, pancreatic carcinoma

## Abstract

Radiotherapy (RT) is a commonly employed treatment in oncological setting. Unfortunately, the therapeutic dose required for tumor control often induces significant side effects in normal tissues, leading to suboptimal outcomes and reduced patient quality of life. FLASH radiotherapy (FLASH-RT) is distinguished by its exceptionally high dose rates, surpassing 40 Gy/s. This advancement has emerged as a promising innovation in the field of cancer treatment. Indeed, FLASH-RT may reduce normal tissue toxicity compared to conventional radiotherapy (CONV-RT) while maintaining tumor control. Here, FLASH-RT and CONV-RT have been compared in an alternative embryonic model of pancreatic carcinoma, a cancer type with poor prognosis and limited therapeutic options. The chorioallantoic membrane models (CAMs) have been employed to assess the tumor control and the treatment toxicity by analyzing the embryo survival. FLASH-RT exhibited significantly reduced off-target toxicity on the embryos, as evidenced by the embryonic growth analysis and histopathological analysis. The tumor control outcomes were comparable between FLASH-RT and CONV-RT, confirming the iso-efficacy between the two strategies. These findings confirm the paradigm-shifting potential of FLASH-RT to enhance the therapeutic ratio, particularly in anatomically complex and radioresistant tumors such as pancreatic carcinoma, warranting further investigation in clinical settings. Additionally, a solid embryonic *in vivo* model has been introduced for comprehensive investigations on emerging radio-treatment approaches. While further investigations are necessary to optimize dose delivery and evaluate long-term outcomes, this research underscores the transformative promise of FLASH-RT in redefining radiotherapy standards for challenging malignancies.

## Graphical abstract

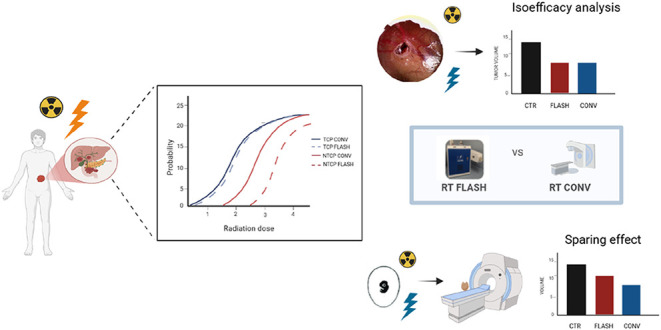

## 1 Introduction

Radiotherapy (RT) is a commonly employed treatment in oncological setting despite the therapeutic dose required to eradicate the tumor sometimes overlaps with the one that causes significant toxicity to normal tissues.^1^ This overlap limits the treatment efficacy and may lead to both loco-regional recurrence and a reduced patient’s quality of life.^2^ Advanced RT techniques, such as intensity-modulated RT (IMRT), image-guided RT (IGRT), and stereotactic body RT (SBRT), have increased the treatment accuracy for the management of some carcinoma in areas such as the thorax, head and neck, and pelvis.^3^ Unluckily, despite these advancements, the effectiveness of these RT strategies is still limited to treat aggressive cancers such as pancreatic adenocarcinoma (PAC).

PAC is the malignancy with the lowest five-year survival rate (9%) among all major cancers because of its aggressive biology, vague early symptoms, and the inherent difficulties in early detection.^4^ More than half of PAC cases are diagnosed at an advanced stage, when metastasis has already occurred, reducing the five-year survival rate to 2.9%.^5^ RT may be the game changer in the PAC treatment if an effective therapeutic dose can be delivered on the tumor while minimizing the toxicity to surrounding tissues. Indeed, the site of the pancreas is in the upper abdomen, and it is surrounded by critical structures particularly susceptible to radiation damage, such as the rapidly dividing cells of the gastrointestinal (GI) tract.^6,7^ In clinical settings, PAC requires high radiation doses (>50 Gy) to be effectively treated, leading to intolerable toxicities. Indeed, acute and late GI complications often arise.^8^ The radiosensitivity of tissues adjacent to pancreas, including the intestine, liver, and stomach, contribute to the risk of acute adverse effects such as mucosal lesions and inflammation. Chronic complications may also occur, including fibrosis, vascular sclerosis, biliary stricture, chronic diarrhea, malabsorption, small intestinal obstruction, ulceration, and hemorrhage.^9,10^ Thus, introducing a technique that mitigates the radiation toxicity while maintaining therapeutic efficacy for the treatment of PAC would result in a cornerstone in oncology.

In this context, FLASH-RT represents a promising strategy for the treatment of PAC. FLASH-RT is an innovative technique in which the radiation is administered at ultrahigh dose rates (≥40 Gy/s) in extremely short periods (100–200 ms).^11^ Some investigations, among which the seminal study by Favaudon et al., have indicated the *in vivo* tissue-sparing benefits (FLASH effect) respect to CONV-RT with brain, breast, lung, head and neck, skin, and ovarian cancers.^12,13^ Despite the accumulating evidence supporting the FLASH effect, the underlying radiobiological mechanisms remain largely elusive. Emerging data suggest that multiple processes may operate in concert, encompassing radiolytic oxygen depletion (ROD), modulation of reactive oxygen species (ROS) generation and recombination, alterations in DNA damage response, vascular and immune modulation, and changes in redox homeostasis.^14^ While these hypotheses hold significant interest, their simultaneous and dynamic interplay presents challenges in isolating them experimentally, primarily due to the limited availability of animal models capable of capturing all these aspects in an integrated manner. Most studies so far have focused on adult animal models, with zebrafish embryos being a notable exception. In 2018, Gruel et al. investigated the effects of FLASH-RT on zebrafish embryos, demonstrating that developing biomodels are an appropriate platform for investigating RT effects.^15^ This is because the dynamic molecular environment of embryonic tissues amplifies the interactions between radiation and cells, making them an ideal model for studying RT effects. Indeed, active cell replication enhances the susceptibility of cells to radiation-induced damage, facilitating the investigation of the radiation effects on pivotal processes such as DNA replication, damage response, and tissue remodeling.^16^ Thus, embryos are an advantageous platform to shed light on the mechanisms behind the FLASH effects, accelerating the identification of critical factors for the clinical optimization of this technique. Regrettably, despite the user-friendly nature, zebrafish models are constrained by their anatomical and physiological simplicity.^17^

To address this gap, this work introduces the chorioallantoic membrane (CAM) chicken model as a robust embryonic biomodel to investigate both the tissue-sparing effect and the treatment efficacy of FLASH-RT in comparison to CONV-RT by utilizing our established protocols. The CAM offers numerous benefits in preclinical oncology due to its versatile and effective platform for *in vivo* tumor investigations, emerging therapies evaluation, and antimetastatic agents’ validation.^18–20^ It provides a cost-effective and ethical alternative to animal studies, reducing the reliance on mammalian models.^21^ By exploiting CAMs, we establish that FLASH-RT substantially diminishes the toxicity of the treatment on the healthy tissues of the embryo compared to CONV-RT. Concurrently, we validate the *in vivo* iso-efficacy between the two radiation strategies in the treatment of pancreatic carcinoma, thereby suggesting the potential applicability of FLASH-RT for the treatment of challenging tumors such as pancreatic adenocarcinoma (PAC) in various clinical settings, including locally advanced disease and the adjuvant context.

## 2 Materials and Methods

### 2.1 Embryonic Development Model

Fertilized red Leghorn eggs were purchased from a local supplier and immediately stored at 4 °C. Before incubation, at Embryonic Day of Development (EDD) 0, the eggs were cleaned with deionized water and placed in trays inserted in a fan-assisted incubator (FIEM MG 140/200) set at 37.5 °C with ≈47% humidity. The experiment began with egg incubation on EDD0, followed by windowing the eggshell on EDD3. The first MRI scan was performed on EDD6, after which embryos were subjected to either FLASH or CONV-RT by using an Electron FLASH linear accelerator (linac SIT). Embryos were irradiated with a total dose of 8 Gy. Subsequent MRI scans were conducted on EDD10 and 16 to track developmental progress. A clinical contouring system (Eclipse system) has been employed to delineate the embryos and allowing to extract the volumetric data. On EDD16 the experiments were concluded, and organs harvested for following biological end-point analysis.

### 2.2 Magnetic Resonance Imaging

MRI image was carried out with a 1.5 T MRI scanner (General Electric Healthcare, Milwaukee, WI) present at the radiology department of the Pisa hospital using a clinical head coil. After the acquisition of triplane conventional Localizer images, a 4-chamber localizer view was acquired using a single-shot steady-state free precession sequence (SSFP). Then, a 3D-Fat Sat prepared SSFP pulse sequence was acquired with the following parameters in axial and in coronal planes: slab thickness 9 cm, slice thickness of 0.9 mm (1.8 mm interpolated), no gap, FOV 22 cm × 22 cm, phase FOV 1, matrix 256 × 256, reconstruction matrix 512 × 512, flip angle of 45°, and a TR/TE ratio approximated = 2.

### 2.3 Cell Culture

Human pancreatic cancer cell line BxPC-3 (ATCC CRL-1687) was purchased from American Type Culture Collection (ATCC). BxPC-3 cells were cultured in RPMI 1640 medium (Gibco 11875093) supplemented with 10% FBS, 1 mm sodium pyruvate (Gibco 11360070), and 1× penicillin–streptomycin (equivalent to 50 U mL^–1^; Gibco 15140–122). Cells were maintained in an incubator set at 37 °C and 5% CO2.

### 2.4 Clonogenic Assay

An increasing number of cells according to the selected irradiation doses (2, 4, 8, and 12 Gy) has been seeded. Twenty-four h after seeding, the plates were irradiated and subsequently monitored for up to 14 days to let the formation of clones. After the incubation period, the cells were washed, fixed and stained with 6% crystal violet solution (Thermo scientific, Cat: 405830250) diluted in ethanol. The number of colonies was determined by manual counting. The survival fraction (SF) was determined by dividing the plating efficiency (PE) of the treated cells by the PE of the corresponding nontreated cells. PE was calculated by dividing the number of colonies formed by the number of cells seeded. Data is reported as mean ± SD of three independent experiments performed in duplicate.

### 2.5 Chorioallantoic Membrane Tumor Model

CAM models of pancreatic carcinoma have been produced through standard protocols.^22^ The preincubation phase for the eggs follows the previously described protocol, with the same conditions for humidity and temperature maintained during incubation. Fertilized red Leghorn eggs were punctured on EDD3 and a specific amount of BxPC3 cells (3 × 10^6^) inoculated on the CAM at EDD6. At EDD10, 4 days postgrafting, eggs were randomized and divided into 2 different treatment groups: (i) FLASH-RT; and (ii) CONV-RT, both receiving a total dose of 2 Gy. On EDD15, the experiment was concluded, and tumors were harvested for following analysis. Tumors were photographed and monitored until EDD15, and their dimensions were measured using a portable digital microscope (DinoLite). The volumes were derived using the formula 1/2 (length × width^2^), where the length and width corresponded to the largest and smallest diameter, respectively. Thirty-eight eggs were used: 13 in the control group, 13 in the CONV group, and 12 in the FLASH group.

### 2.6 Hystological Analysis

Liver was fixed in 10% buffered formalin. Formalin-fixed, paraffin-embedded samples from the three study subgroups (CONV, FLASH and CTR) were sectioned at 4 μm using a microtome and mounted onto microscope slides. Sections were stained with Masson’s trichrome to visualize the fibrotic component and connective tissue distribution. Finally, sections were dehydrated through graded alcohols, cleared in xylene, and mounted with a synthetic mounting medium. Collagen fibers appeared blue (or green), muscle and cytoplasm red, and nuclei blue-black. Slides were subsequently digitized at high resolution using the Aperio AT2 scanner (Leica). Images were acquired in. svs format. The Aperio AT2 system allows initial imaging of the sample using the “*Snapshot*” function, enabling selection of the region of interest. After obtaining a preliminary image, the desired magnification (40×) was selected for scanning, producing high-resolution digital images.

Digitized images were uploaded and analyzed using the image analysis software HALO (Indica Laboratories). A tissue classification algorithm (Area Quantification v2.4.2, Indica Laboratories) was developed based on three chromatic classes: blue for the identification of connective tissue (collagen fibers), red for nonconnective tissue components (muscle fibers, cytoplasm, and cellular structures) and black for nuclei. Representative regions of collagen (blue), nonconnective tissue (red), and nuclei (black) were manually annotated on the digitized images to train the algorithm. The trained algorithm was subsequently applied to all samples, enabling automated identification and quantification of collagen areas. Results were normalized by expressing the collagen content as the percentage of the area occupied by collagen relative to the total tissue area analyzed for each sample.

### 2.7 Morphometric Analysis

The length of the third toe was selected as the anatomical reference for growth assessment, given its reliability and relevance within the selected developmental window. Measurements were performed using a calibrated ruler, extending from the distal tip of the toe to the midpoint of the metatarsophalangeal joint.

### 2.8 *In Vitro* and *In Vivo* Irradiation

Irradiation was performed with an ElectronFLASH accelerator for preclinical studies (Sordina IORT Technologies S.p.A., Aprilia, Italy)^23^ under normoxic conditions at room temperature with the plates lying flat and irradiated from beneath (beam angle 180°). This linear accelerator is capable of delivering electron beams with nominal energies of 7 or 9 MeV and offers a high degree of flexibility in beam parameter control: the electron beam current can be changed from 1 to 100 mA, the pulse duration can vary from 0.5 to 4 μs and the pulse repetition frequency (PRF) can be changed from 1 to 245 Hz without modifying the energy spectrum of the beam. This flexibility makes it possible to vary both the average dose rate (ADR) and the dose per pulse (DPP) independently and switch between conventional (CONV) and FLASH irradiation modalities without the need to modify the experimental setup, making it ideal for comparative studies.^24^ A detailed summary of the irradiation parameters used in this work is provided in Table 1.

**Table 1:** This Table Summarizes the Relevant Physics Parameters Used in Each Irradiation Condition

irradiation modality	dose per pulse	average dose rate	average dose within a pulse	pulse duration
FLASH	1 Gy	240 Gy/s	2.5 × 10^5^ Gy/s	4 μs
CONV	0.02 Gy	6 Gy/min	5 × 10^3^ Gy/s	4 μs

For all the in vitro experiments, both conventional (CONV) and FLASH irradiations were conducted using the Ø100 mm applicator with electrons of 9 MeV (quality indices [56] *R*_50_ = 37 mm, *E̅*_0_ = 8.62 MeV). This configuration ensured a highly uniform dose distribution, with homogeneity higher than 95% across the entire surface area of the irradiated cells. This allows a minimization of the spatial variability in the delivered dose. The linear accelerator operated in the vertical configuration with the Petri dishes positioned atop a 12 mm thick solid water slab, corresponding to the build-up depth for the energy employed. In this work, the PRF was changed to maintain a constant average dose rate (ADR) of 240 Gy/s for all the different dose conditions in these experiments. This value significantly exceeds the commonly reported threshold of 100 Gy/s required to trigger the FLASH effect, and it can be preserved regardless of variations in the total delivered dose. The relationship between the ADR and beam parameters can be described by the following expression, assuming *n* delivered pulses and neglecting the temporal width of each pulse

$$\mathrm{ADR}=\frac{n}{n-1}\cdot \mathrm{PRF}\cdot \mathrm{DPP}$$

The dosimetric characterization of our beams was performed using the flash Diamond (fD) detector, and other dosimeters and techniques.^25–34^ The beam output variations were considered using the beam onitoring system based on the ACCT (Bergoz Instrumentation, Saint-Genis-Pouilly, France).^23^

### 2.9 *In Vivo* Irradiation

To optimize precision, the embryos were positioned on a PMMA disc in front of the accelerator collimator, and irradiation was delivered in two opposing fields, with the egg being rotated between fields (Figure S1). Given the impossibility of achieving acceptable dose coverage in the target zone (where the embryo lies) due to the egg’s dimensions using a single field, two opposing fields were necessary. We evaluate the dose distribution with a single field and with two opposite fields using a Monte Carlo simulation. (Figure S2) The same parameters shown in Table 1 were used for all *in vivo* irradiations.

### 2.10 Statistical Analysis

The data were reported as mean ± standard deviation (SD). Group comparisons were performed using one-tailed Student’s *t* tests and two-way analysis of variance (ANOVA), as indicated in the figure captions. For the *in vivo* embryo and tumor growth delay data, mean values, and their corresponding standard errors of the mean (SEM) were calculated for each group. Differences between groups were evaluated using the Tukey’s multiple comparisons test. Regarding the *in vivo* survival data, the median survival for each group was determined, and differences between groups were assessed using the log-rank test. Histological analyses were statistically evaluated using the Mann–Whitney and Wilcoxon tests for nonparametric comparisons, and one-way ANOVA for multiple group comparisons. Statistical significance was defined as *p* < 0.05. All statistical analyses were conducted using GraphPad Prism software (GraphPad Software Inc., La Jolla, CA).

## 3 Results and Discussion

CAMs have been employed for the simultaneous analysis of the radiation effects on the embryo and on the primary tumor to assess the impact of FLASH radiotherapy on both the overall toxicity and tumor control in comparison to conventional radiotherapy. To the best of our knowledge, this is the very first comparative analysis exploiting embryonic chicken models with electron FLASH. It should be highlighted that CAMs exhibit highly radiosensitive characteristics in terms of cell proliferation, differentiation, and migration.^35–39^ These pivotal features make them crucial for anticipating the potential radiation risks in humans under clinically relevant conditions. The efficacy comparison has been preliminarily assessed *in vitro* by using pancreatic cancer cells (BxPC-3) for clonogenic assays. After 14 days, the ability of cells to form colonies following radiation exposure revealed no significant differences between cells exposed to FLASH-RT or CONV-RT at any of the tested doses (Figure S3). Based on these results, we further compared the effect of the two radiation strategies in vivo models. BxPC3 cells were implanted on the CAM at embryonic day of development (EDD) 6. After 4 days (EDD10) the vascularized tumors were irradiated with 2 Gy for both FLASH and CONV modalities (Figure 1A). This dosage has been selected because, in accordance with our previous research, it is the most effective for evaluating the impact of radiotherapy on pancreatic tumor volume in CAMs.^19,22^ Indeed, it enables the precise quantification of the tumor size reduction while avoiding significantly compromises the embryo viability. Moreover, from a technical perspective, 2 Gy represents the minimum dose required to ensure an adequate irradiation of the chick model using two distinct irradiation fields, considering the physical constraints of dose delivery. After the irradiation, the tumor growth was daily monitored until EDD15. The tumor volume analysis showed a significative reduction compared to the control in both the treated groups. No statistically significant differences were found between FLASH-RT and CONV-RT, suggesting the iso-efficacy between the two treatment modalities (Figure 1B, and S4).

**Figure 1:**
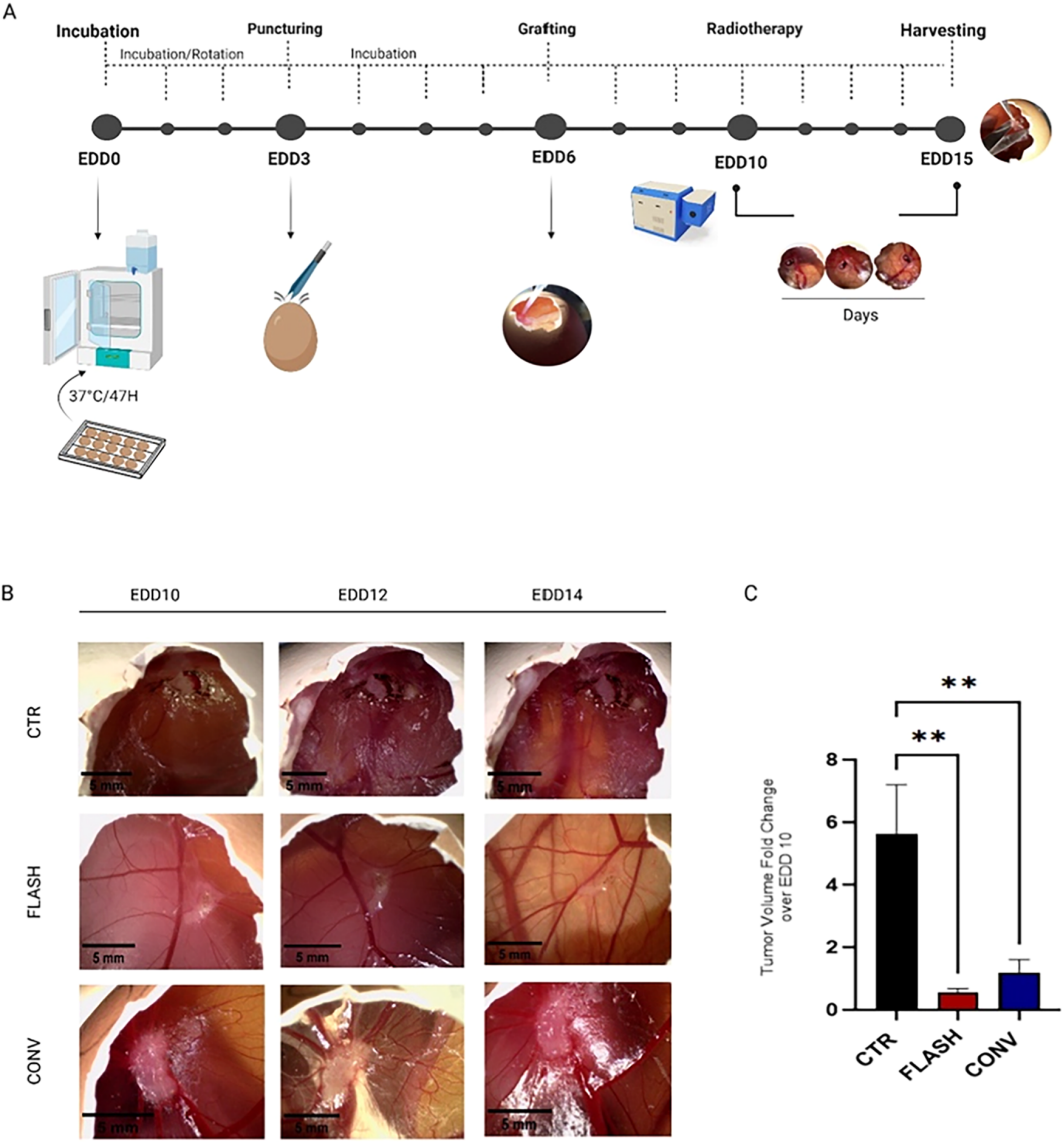
(A) Schematic representation of the experimental setup. On embryonic day of development 6 (EDD6), 3 × 10^6^ BX-PC3 cells were grafted on the chorioallantoic membrane (CAM). Radiotherapy (RT) was administered on EDD10, and tumor growth was monitored until EDD15, when tumors were collected for analysis. (B) Representative images of BX-PC3 xenograft tumors of the different treatment conditions (CTR, FLASH, CONV) (Scale bar: 5 mm) (CONV-RT vs CTR *p* = 0.0069; FLASH-RT vs CTR *p* = 0.0023). (C) Tumor volume analysis at EDD15. Sample size *n* = 38. Both FLASH and CONV treatments significantly reduced tumor size compared to the untreated control (*p* < 0.01).

The sparing effect of FLASH-RT has been evaluated by irradiating the embryos with a dose of 8 Gy at EDD6 and monitoring their volume by magnetic resonance imaging (MRI) at EDD6, 10, and 16 (Figure 2A). Eight Gy has been selected as it provides optimal conditions for influencing embryonic development while simultaneously exerting a measurable impact on viability. The irradiation has been administered at EDD6 to assess the treatment during the more vulnerable developmental phase and to extend the evaluation period as far as possible. In all irradiation conditions (Figure 2B), a decrease in survival has been observed relative to the control group. However, this impact has been more pronounced for the embryos of the CONV-RT group by reaching the 40% of overall survival with respect to the 58% recorded in the FLASH-RT condition. Together with that, the median embryo survival was significantly lower in the conventional RT group (13 days). Subsequent analyses focused on the assessment of potential embryonic growth impairments due to irradiations (both FLASH and CONV) compared to the control. Embryonic development has been evaluated by MRI and by applying a contouring approach (commonly used in clinical settings) to delineate the embryos profiles and noninvasively evaluate the volume at days 6, 10, and 16 (Figure 2C).

**Figure 2:**
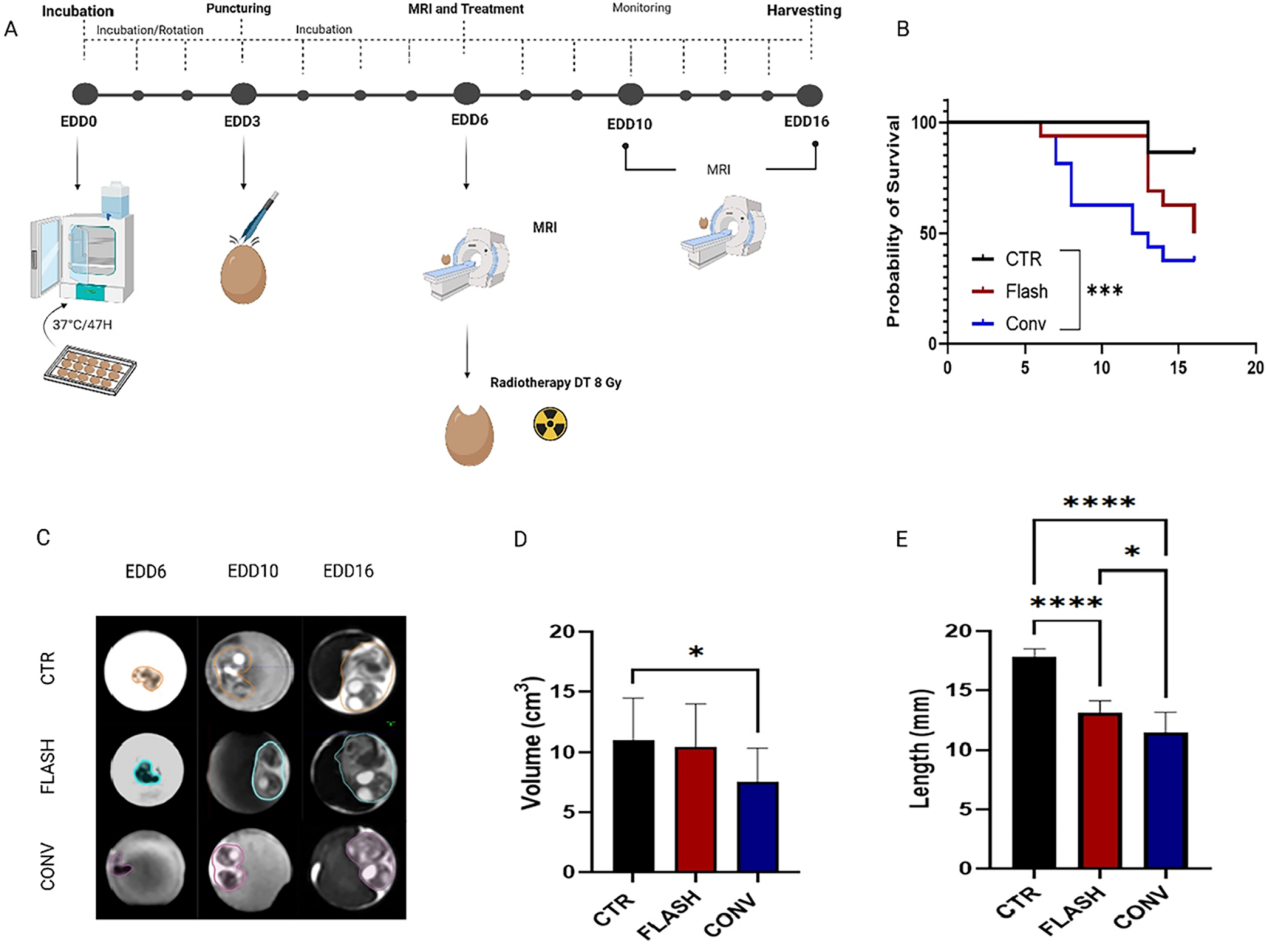
(A) Schematic representation of the experimental protocol. MRI was performed at embryonic day 6 (EDD6), immediately followed by irradiation. MRI scans were acquired at EDD10 and EDD16. At EDD16, organs were collected for histological analysis. (B) Kaplan–Meier survival curves showing embryo viability from EDD10 to EDD16 of the different treatment groups (CTR, FLASH, CONV). A significant improvement in survival was observed in the FLASH group compared to CONV (CTR vs CONV *p* = 0.0004, CTR vs FLASH *p* = 0.0485, FLASH vs CONV *p* = 0.063). (C) Representative MRI images of chick embryos at EDD6, EDD10, and EDD16 with volumes contoured using Eclipse software. (D) Embryo development was quantified by measuring the total volume (cm^3^) at EDD16 for each group. FLASH-treated embryos showed a significantly greater volumetric growth compared to CONV, while no significant difference was observed between CTR and FLASH. (E) Measurement of the 3rd toe length in control (CTR), FLASH-RT, and CONV-RT embryos. CTR embryos exhibited the greatest digit length, while a significant reduction was observed in both irradiated groups (CTR vs CONV-RT, *p* = < 0,0001; CTR vs FLASH-RT, *p* = < 0,0001; FLASH-RT vs CONV-RT, *p* = 0,0387). Notably, FLASH-treated embryos retained a significantly greater length compared to CONV-treated counterparts, suggesting a protective effect of FLASH-RT on distal limb development. Data are reported as mean ± SEM of three independent experiments, each with at least 5 embryos per condition. Statistical significance: *p* < 0.05 (*), *p* < 0.0001 (**).

This innovative approach facilitates a precise quantitative assessment of embryonic growth, enabling the analysis of the sparing effect. Notably, MRI has previously been utilized on CAMs to analyze tumor growth, cell migration, and drug response rather than directly evaluating radiotherapy-induced toxicity.^40–42^ For instance, Oppitz et al. investigated melanoma cell migration using MRI, demonstrating its utility in tracking the migration patterns of transplanted tumor cells.^41^ Additionally, other studies demonstrated that the novel chimeric inhibitor Animacroxam effectively reduces testicular tumor growth and angiogenesis, utilizing this imaging tool.^40^ The volumes collected at EDD16 were normalized over the corresponding values recorded at EDD6 for each group. Embryos irradiated with CONV-RT showed a significant growth reduction compared to control group (CTR vs CONV *p*-value = 0.0177). On the other hand, no significant development alteration was observed in the FLASH-RT group compared to controls, further confirming the tissue-sparing effect of FLASH-RT (Figure 2D). The analysis of volume progression across all time points (days 6, 10, and 16) reinforces these findings, revealing a statistically significant difference between FLASH and conventional irradiation modalities (*p* = 0.0029) (Figure S5).

The experiment was terminated at EDD16. Consistently with the data obtained from the contouring system, the embryos irradiated with CONV-RT were visually smaller in size compared to the nonirradiated group and showed visible signs of tissue toxicity, including alopecia and edema (Figure S6), further confirming the detrimental effects of CONV-RT on tissue integrity. The two irradiation strategies were further evaluated by analyzing the digit III (3rd toe). The third toe has been selected as a reference structure due to its central anatomical position, well-defined morphology, and representativeness of the limb’s proliferative activity during development.^43^ This is supported by Wolpert’s Progress Zone model and established concepts of limb morphogenesis, which indicate that by embryonic day 6, positional identities are defined, and further limb outgrowth is predominantly sustained by mesenchymal proliferation.^44,45^ The quantitative analysis (Figure 2E) demonstrated that, while both irradiated groups exhibited a substantial reduction in digit III length relative to controls, embryos subjected to FLASH irradiation exhibited significantly enhanced preservation of distal limb growth compared to those treated with CONV-RT, further validating the protective effect of FLASH-RT. Overall, FLASH-RT better preserves the cellular integrity and tissue-level signaling compared to CONV-RT, as demonstrated by the partial preservation of the digit III growth.

Finally, the organs of the embryos were collected to investigate the tissues by histological analysis. The percentage of collagen relative to the total tissue area, assessed by Masson’s trichrome staining in embryos liver samples, was quantified across three experimental groups: FLASH (*n* = 6), CONV (*n* = 6), and control (CTR, *n* = 4). None of the three comparisons revealed statistically significant differences. A one-way ANOVA was subsequently conducted to compare all three groups simultaneously, which also did not show significant differences (*p* = 0.5744). Despite the absence of statistical significance, the data revealed a trend in which the CONV group exhibited higher percentages of collagen compared to FLASH and CTR, while the latter two groups showed comparable values. Some preclinical studies suggested that FLASH radiotherapy reduces fibrosis formation, a side effect impacting tumor treatment and surgical outcomes.^13,46^ Thus, the histological analyses performed on liver tissue sections tended toward reduced collagen accumulation following FLASH-RT (Figure 3) suggesting a potential benefit of this approach for PAC management, in which gastrointestinal toxicities limit the effectiveness of the treatment by impairing dose escalation, treatment adherence, feasibility, and quality of life.^47–49^

**Figure 3:**
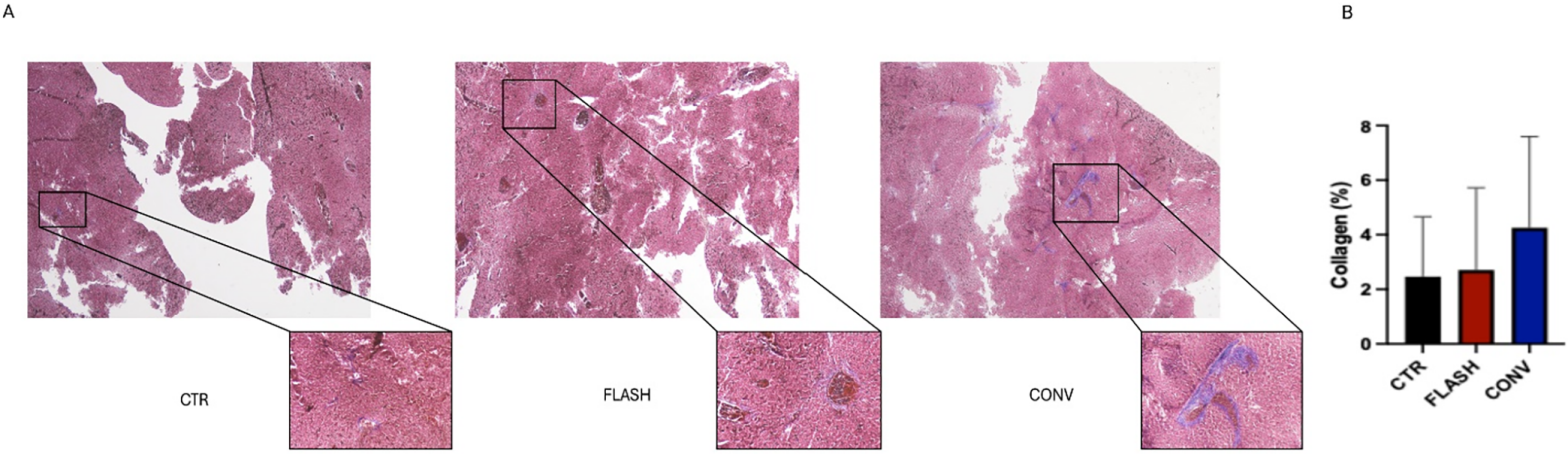
(A) Representative Masson’s trichrome-stained liver sections of control (CTR), FLASH-RT, and CONV-RT embryos. Blue staining indicates collagen deposition and fibrotic areas. (B) Descriptive analysis showed that CONV samples displayed collagen percentages ranging from 0.6702 to 8.328, with a median value of 3.849 and a mean of 4.256. FLASH samples ranged from 0.0000 to 7.395, with a median of 1.769 and a mean of 2.708. CTR samples showed collagen values ranging from 1.233 to 5.757, with a median of 1.433 and a mean of 2.464. To improve data consistency and reliability, outliers were previously identified and excluded, resulting in the removal of one sample per group. As the data did not follow a normal distribution, nonparametric tests were applied. Statistical analyses (Mann–Whitney, Wilcoxon, and one-way ANOVA) did not reveal significant differences among groups (CONV vs FLASH) (*p* = 0.3701, Mann–Whitney; *p* = 0.1562, Wilcoxon), CONV vs CTR (*p* = 0.2571, Mann–Whitney; *p* = 0.6250, Wilcoxon), FLASH vs CTR (*p* > 0.9999, Mann–Whitney; *p* = 0.2500, Wilcoxon). However, the data indicated a pattern in which the CONV group tended to exhibit higher collagen percentages, whereas FLASH and CTR showed comparable values.

## 4 Conclusions

Overall, our results on iso-efficacy and sparing effect suggest that FLASH-RT may be particularly beneficial for nonresectable and borderline resectable pancreatic tumors (approximately one-third of pancreatic cancer patients), in which expanding the therapeutic window is crucial due to the high radiation doses required for effective tumor control. This is particularly relevant by considering the recent results from the phase 3 PREOPANC study, which demonstrated that a relatively low preoperative dose of radiotherapy (36 Gy in 15 fractions) combined with gemcitabine led to an increase in postoperative complications compared to chemotherapy alone (68% vs 50%, *P* = 0.026).^50,39^ Although the addition of radiotherapy improved local control (R0 resection rates: 71% vs 40%) and median overall survival (from 13.2 months to 17.6 months, *P* = 0.029), the associated toxicity remains a significant concern, especially in the context of routine clinical practice. Moreover, FLASH-RT may be also applied in adjuvant settings for sterilizing residual disease foci in anatomically challenging regions like the retroperitoneal space. Tumor recurrence often involves perineural invasion (PNI), a hallmark of PAC linked to poorly controlled pain.^51^ Pancreatic cancer exhibits the highest prevalence of PNI among solid tumors, reported in 70–98% of cases, underscoring its pervasive and aggressive nature. The association between PNI and pain is well-documented in the literature.^52,53^ Cancerous invasion disrupts the neural sheath, leading to increased neural density and remodeling, which contributes to both neuropathic and inflammatory pain.^54,55^ These findings highlight the clinical significance of preventing PNI in this cancer to improve patient outcomes. A critical drawback addressed in this study concerns the “time delay” between sequential field irradiations, a technical issue relevant to FLASH-RT with multiple fields. Although in our setup a 1 min delay did not appear to compromise the FLASH effect, the relationship between delivery timing, dose rate, and beam parameters remains poorly understood and warrants further investigation. Noticeably, within this study, we have also introduced the chick embryo model integrated with MRI imaging as a novel, rapid, and cost-effective *in vivo* platform specifically adapted to study FLASH-RT, allowing for real-time observation of both developmental and tumor responses. This represents a significant methodological innovation in the field of radiobiology.

## 5 Limitation of the Study

It should be noted that despite its promise, the clinical translation of FLASH-RT remains limited by current technological constraints.^12,56^ The availability of compact, reliable systems for intraoperative use is still under development, and deep-seated tumor targeting with electrons is hindered by the lack of clinically viable Very High Energy Electron (VHEE) platforms. These limitations currently prevent routine implementation in deep tumors, suggesting that intraoperative FLASH-RT represents the most feasible near-term application. Future research should focus on three key priorities: (i) optimizing beam delivery strategies and timing to preserve the FLASH effect in multifield protocols; (ii) developing robust dose distribution and treatment planning tools compatible with FLASH-RT; and (iii) extending biological investigations to include gene expression profiling and immunomodulatory effects in immunocompetent biomodels. Indeed, although CAMs represent *in vivo* models that are ideally suited for research on FLASH-RT, the observation window to assess treatment response is limited to approximately 10 days, preventing the evaluation of radiation-induced late toxicity. Moreover, the absence of a fully developed immune system at the early stages of embryonic development may limit the investigation of immune-mediated and inflammatory processes.

## Supplementary Material



## Data Availability

All data generated and analyzed during this study are included in this published article and its Supporting Information files. The raw data are available from the corresponding author upon reasonable request.

## Notes

Irradiation data (experimental setup, dosimetric evaluation), clonogenic results, tumor volume fold change, embryo volume development curve, embryo toxicity (PDF)

## References

[1] Marks L. B., Yorke E. D., Jackson A., Ten Haken R. K., Constine L. S., Eisbruch A., Bentzen S. M., Nam J., Deasy J. O. (2010). Use of Normal Tissue Complication Probability Models
in the Clinic. Int. J. Radiat. Oncol. Biol.
Phys..

[2] Montay-Gruel P., Meziani L., Yakkala C., Vozenin M. C. (2019). Expanding the Therapeutic
Index of Radiation Therapy by Normal Tissue Protection. Br. J. Radiol..

[3] Thariat J., Hannoun-Levi J. M., Myint A. S., Vuong T., Gérard J. P. (2013). Past, Present,
and Future of Radiotherapy for the Benefit of Patients. Nat. Rev. Clin. Oncol..

[4] Khalaf N., El-Serag H. B., Abrams H. R., Thrift A. P. (2021). Burden of Pancreatic
Cancer: From Epidemiology to Practice. Clin.
Gastroenterol. Hepatol..

[5] World Cancer Research Fund. . Pancreatic Cancer Statistics. https://www.wcrf.org/preventing-cancer/cancer-statistics/pancreatic-cancer-statistics/. (Accessed January 15, 2025).

[6] Shadad A. K., Sullivan F. J., Martin J. D., Egan L. J. (2013). Gastrointestinal
Radiation Injury: Symptoms, Risk Factors and Mechanisms. World J. Gastroenterol..

[7] Kim S. K., Wu C. C., Horowitz D. P. (2016). Stereotactic
Body Radiotherapy for
the Pancreas: A Critical Review for the Medical Oncologist. J. Gastrointest. Oncol..

[8] Falco M., Masojć B., Sulikowski T. (2023). Radiotherapy in Pancreatic Cancer:
To Whom, When, and How?. Cancers.

[9] Lu Q., Liang Y., Tian S., Jin J., Zhao Y., Fan H. (2023). Radiation-Induced Intestinal Injury:
Injury Mechanism and Potential
Treatment Strategies. Toxics.

[10] Elhammali A., Patel M., Weinberg B., Verma V., Liu J., Olsen J. R., Gay H. A. (2015). Late Gastrointestinal
Tissue Effects
after Hypofractionated Radiation Therapy of the Pancreas. Radiat. Oncol..

[11] Schüler E., Acharya M., Montay-Gruel P., Loo B. W., Vozenin M. C., Maxim P. G. (2022). Ultra-High
Dose Rate Electron Beams
and the FLASH Effect: From Preclinical Evidence to a New Radiotherapy
Paradigm. Med. Phys..

[12] Giannini N., Gadducci G., Fuentes T., Gonnelli A., Di Martino F., Puccini P., Naso M., Pasqualetti F., Capaccioli S., Paiar F. (2024). Electron FLASH Radiotherapy In Vivo
Studies: A Systematic Review. Front. Oncol..

[13] Favaudon V., Caplier L., Monceau V., Pouzoulet F., Sayarath M., Fouillade C., Poupon M. F., Brito I., Hupé P., Bourhis J., Hall J., Fontaine J. J., Vozenin M. C. (2014). Ultrahigh
Dose-Rate FLASH Irradiation Increases the
Differential Response between Normal and Tumor Tissue in Mice. Sci. Transl. Med..

[14] Chow J. C. L., Ruda H. E. (2024). Mechanisms of Action
in FLASH Radiotherapy: A Comprehensive
Review of Physicochemical and Biological Processes on Cancerous and
Normal Cells. Cells.

[15] Montay-Gruel P., Acharya M. M., Petersson K., Alikhani L., Yakkala C., Allen B. D., Ollivier J., Petit B., Jorge P. G., Syage A. R., Nguyen T. A., Baddour A. A. D., Lu C., Singh P., Moeckli R., Bochud F., Germond J. F., Froidevaux P., Bailat C., Bourhis J., Vozenin M. C., Limoli C. L. (2019). Long-Term
Neurocognitive Benefits of FLASH Radiotherapy
Driven by Reduced Reactive Oxygen Species. Proc.
Natl. Acad. Sci. U.S.A..

[16] Valentin J. (2003). Biological
Effects after Prenatal Irradiation (Embryo and Fetus): ICRP Publication
90. Ann. ICRP.

[17] Adhish M., Manjubala I. (2023). Effectiveness
of Zebrafish Models in Understanding
Human Diseases—A Review of Models. Heliyon.

[18] Gonnelli A., Sarogni P., Giannini N., Linsalata S., Di Martino F., Zamborlin A., Frusca V., Ermini M. L., Puccini P., Voliani V., Paiar F. (2024). A Bioconvergence Study
on Platinum-Free Concurrent Chemoradiotherapy for the Treatment of
HPV-Negative Head and Neck Carcinoma. Artif.
Cells, Nanomed., Biotechnol..

[19] Zamborlin A., Sarogni P., Frusca V., Gonnelli A., Giannini N., Ermini M. L., Marranci A., Pagliari F., Mazzanti C. M., Seco J., Voliani V. (2023). Drug-Free
Hybrid Nanoarchitecture
Modulation of the Metastatic Behavior of Pancreatic Ductal Adenocarcinoma
in Alternative In Vivo Models. ACS Appl. Nano
Mater..

[20] Bassi G., Rossi A., Campodoni E., Sandri M., Sarogni P., Fulle S., Voliani V., Panseri S., Montesi M. (2024). 3D Tumor-Engineered
Model Replicating the Osteosarcoma Stem Cell Niche and In Vivo Tumor
Complexity. ACS Appl. Mater. Interfaces.

[21] Mapanao A. K., Che P. P., Sarogni P., Sminia P., Giovannetti E., Voliani V. (2021). Tumor-Grafted Chick Chorioallantoic Membrane as an
Alternative Model for Biological Cancer Research and Conventional/Nanomaterial-Based
Theranostics Evaluation. Expert Opin. Drug Metab.
Toxicol..

[22] Sarogni P., Zamborlin A., Mapanao A. K., Logghe T., Brancato L., van Zwol E., Menicagli M., Giannini N., Gonnelli A., Linsalata S., Colenbier R., Van den Bossche J., Paiar F., Bogers J., Voliani V. (2023). Hyperthermia Reduces
Irradiation-Induced Tumor Repopulation in an In Vivo Pancreatic Carcinoma
Model. Adv. Biol..

[23] Di
Martino F., Del Sarto D., Bass G., Capaccioli S., Celentano M., Coves D., Douralis A., Marinelli M., Marrale M., Masturzo L., Milluzzo G., Montefiori M., Paiar F., Pensavalle J. H., Raffaele L., Romano F., Subiel A., Touzain E., Rinati G. V., Felici G. (2023). Architecture,
Flexibility, and Performance of a Special Electron Linac Dedicated
to FLASH Radiotherapy Research: ElectronFlash with a Triode Gun of
the Centro Pisano FLASH Radiotherapy (CPFR). Front. Phys..

[24] Chow J. C. L., Ruda H. E. (2024). Impact of Scattering Foil Composition
on Electron Energy
Distribution in a Clinical Linear Accelerator Modified for FLASH Radiotherapy:
A Monte Carlo Study. Materials.

[25] Rinati G. V., Felici G., Galante F., Gasparini A., Kranzer R., Mariani G., Pacitti M., Prestopino G., Schüller A., Vanreusel V., Verellen D., Verona C., Marinelli M. (2022). Application
of a Novel Diamond Detector for Commissioning
of FLASH Radiotherapy Electron Beams. Med. Phys..

[26] Di
Martino F., Del Sarto D., Bisogni M. G., Capaccioli S., Galante F., Gasperini A., Linsalata S., Mariani G., Pacitti M., Paiar F., Ursino S., Vanreusel V., Verellen D., Felici G. (2022). A New Solution for
UHDP and UHDR (FLASH) Measurements: Theory and Conceptual Design of
ALLS Chamber. Phys. Med..

[27] Di
Martino F., Del Sarto D., Barone S., Bisogni M. G., Capaccioli S., Galante F., Gasparini A., Mariani G., Masturzo L., Montefiori M., Pacitti M., Paiar F., Pensavalle J. H., Romano F., Ursino S., Vanreusel V., Verellen D., Felici G. (2022). A New Calculation Method for the
Free Electron Fraction of an Ionization Chamber in the Ultra-High-Dose-Per-Pulse
Regimen. Phys. Med..

[28] Di
Martino F., Barca P., Barone S., Bortoli E., Borgheresi R., De Stefano S., Di Francesco M., Faillace L., Giuliano L., Grasso L., Linsalata S., Marfisi D., Migliorati M., Pacitti M., Palumbo L., Felici G. (2020). FLASH Radiotherapy with Electrons: Issues Related to
the Production, Monitoring, and Dosimetric Characterization of the
Beam. Front. Phys..

[29] Del
Sarto D., Masturzo L., Cavalieri A., Celentano M., Fuentes T., Gadducci G., Giannini N., Gonnelli A., Milluzzo G., Paiar F., Pensavalle J. H., Romano F., Di Martino F. (2025). A Systematic Investigation on the
Response of EBT-XD Gafchromic Films to Varying Dose-Per-Pulse, Average
Dose-Rate and Instantaneous Dose-Rate in Electron FLASH Beams. Front. Phys..

[30] Ciarrocchi E., Ravera E., Cavalieri A., Celentano M., Del Sarto D., Di Martino F., Linsalata S., Massa M., Masturzo L., Moggi A., Morrocchi M., Pensavalle J. H., Bisogni M. G. (2024). Plastic Scintillator-Based
Dosimeters
for Ultra-High Dose Rate (UHDR) Electron Radiotherapy. Phys. Med..

[31] Milluzzo G., De Napoli M., Di Martino F., Amato A., Del Sarto D., D’Oca M. C., Marrale M., Masturzo L., Medina E., Okpuwe C., Pensavalle J. H., Vignati A., Camarda M., Romano F. (2024). Comprehensive
Dosimetric Characterization of Novel
Silicon Carbide Detectors with UHDR Electron Beams for FLASH Radiotherapy. Med. Phys..

[32] Gómez F., Gonzalez-Castaño D. M., Gómez Fernández N., Pardo-Montero J., Schüller A., Gasparini A., Vanreusel V., Verellen D., Felici G., Kranzer R., Paz-Martín J. (2022). Development of an Ultra-Thin Parallel Plate Ionization
Chamber for Dosimetry in FLASH Radiotherapy. Med. Phys..

[33] Cova F., Morrocchi M., Fasoli M., Ciarrocchi E., Pensavalle J. H., Gallo S., Cavalieri A., Zhang M., Zhang K., Jia Z., Tonelli M., Di Martino F., Vedda A., Bisogni M. G., Veronese I. (2025). Stem Effect-Free
(Y,Yb)­AG-Based Detectors for Ultra-High Dose Rate Electron Beam Dosimetry. Sens. Actuators, A.

[34] Romano F., Milluzzo G., Di Martino F., D’Oca M. C., Felici G., Galante F., Gasparini A., Mariani G., Marrale M., Medina E. (2023). First
Characterization of Novel Silicon Carbide Detectors with Ultra-High
Dose Rate Electron Beams for FLASH Radiotherapy. Appl. Sci..

[35] Mayer M., Kaiser N., Layer P. G., Frohns F. (2016). Cell Cycle Regulation
and Apoptotic Responses of the Embryonic Chick Retina by Ionizing
Radiation. PLoS One.

[36] Naito M., Harumi T., Minematsu T., Tajima A., Kuwana T. (2007). Effect of
Soft X-Ray Irradiation on the Migratory Ability of Primordial Germ
Cells in Chickens. Br. Poult. Sci..

[37] Wolpert L., Tickle C., Sampford M., Lewis J. H. (1979). The Effect of Cell
Killing by X-Irradiation on Pattern Formation in the Chick Limb. Development.

[38] Galloway J. L., Delgado I., Ros M. A., Tabin C. J. (2009). A Reevaluation of
X-Irradiation-Induced Phocomelia and Proximodistal Limb Patterning. Nature.

[39] Goff R. A. (1962). The Relation
of Development Status of Limb Formation to X-Radiation Sensitivity
in Chick Embryos. I. Gross Study. J. Exp. Zool..

[40] Herrmann A., Taylor A., Murray P., Poptani H., Sée V. (2018). Magnetic Resonance
Imaging for Characterization of a Chick Embryo Model of Cancer Cell
Metastases. Mol. Imaging.

[41] Oppitz M., Pintaske J., Kehlbach R., Schick F., Schriek G., Busch C. (2007). Magnetic Resonance Imaging of Iron-Oxide
Labeled SK-Mel-28 Human
Melanoma Cells in the Chick Embryo Using a Clinical Whole-Body MRI
Scanner. Magn. Reson. Mater. Phys., Biol. Med..

[42] Steinemann G., Dittmer A., Schmidt J., Josuttis D., Fähling M., Biersack B., Beindorff N., Koziolek E. J., Schobert R., Brenner W., Müller T., Nitzsche B., Höpfner M. (2019). Antitumor
and Antiangiogenic Activity of the Novel Chimeric Inhibitor Animacroxam
in Testicular Germ Cell Cancer. Mol. Oncol..

[43] Wolpert L. (1999). Vertebrate
Limb Development and Malformations. Pediatr.
Res..

[44] Pickering J., Rich C. A., Stainton H., Aceituno C., Chinnaiya K., Saiz-Lopez P., Ros M. A., Towers M. (2018). An Intrinsic Cell Cycle
Timer Terminates Limb Bud Outgrowth. eLife.

[45] Wolpert L. (2002). The Progress
Zone Model for Specifying Positional Information. Int. J. Dev. Biol..

[46] Bley C. R., Wolf F., Jorge P. G., Grilj V., Petridis I., Petit B., Böhlen T. T., Moeckli R., Limoli C., Bourhis J., Meier V., Vozenin M. C. (2022). Dose- and Volume-Limiting
Late Toxicity of FLASH Radiotherapy in Cats with Squamous Cell Carcinoma
of the Nasal Planum and in Mini Pigs. Clin.
Cancer Res..

[47] Grossberg A. J., Chu L. C., Deig C. R., Fishman E. K., Hwang W. L., Maitra A., Marks D. L., Mehta A., Nabavizadeh N., Simeone D. M., Weekes C. D., Thomas C. R. (2020). Multidisciplinary Standards of Care and Recent Progress
in Pancreatic
Ductal Adenocarcinoma. Ca-Cancer J. Clin..

[48] Loi M., Magallon-Baro A., Suker M., Van Eijck C., Hoogeman M., Nuyttens J. J. (2020). Daily Dose
to Organs at Risk Predicts
Acute Toxicity in Pancreatic Stereotactic Radiotherapy. Acta Oncol..

[49] Henry A. M., Ryder W. D., Moore C., Sherlock D. J., Geh J. I., Dunn P., Price P. (2008). Chemoradiotherapy for
Locally Advanced
Pancreatic Cancer: A Radiotherapy Dose Escalation and Organ Motion
Study. Clin. Oncol..

[50] Versteijne E., Suker M., Groothuis K., Akkermans-Vogelaar J. M., Besselink M. G., Bonsing B. A., Buijsen J., Busch O. R., Creemers G. M., van Dam R. M., Eskens F. A. L. M., Festen S., de Groot J. W. B., Koerkamp B. G., de Hingh I. H., Homs M. Y. V., van
Hooft J. E., Kerver E. D., Luelmo S. A. C., Neelis K. J., Nuyttens J., Paardekooper G. M. R.
M., Patijn G. A., van der Sangen M. J.
C., de Vos-Geelen J., Wilmink J. W., Zwinderman A. H., Punt C. J., van Eijck C. H., van Tienhoven G. (2020). Dutch Pancreatic Cancer Group. Preoperative Chemoradiotherapy
versus Immediate Surgery for Resectable and Borderline Resectable
Pancreatic Cancer: Results of the Dutch Randomized Phase III PREOPANC
Trial. J. Clin. Oncol..

[51] Garajová I., Giovannetti E. (2024). Targeting
Perineural Invasion in Pancreatic Cancer. Cancers.

[52] Liebl F., Demir I. E., Mayer K., Schuster T., D’Haese J. G., Becker K., Langer R., Bergmann F., Wang K., Rosenberg R., Novotny A. R., Feith M., Reim D., Friess H., Ceyhan G. O. (2014). The Impact of Neural Invasion Severity
in Gastrointestinal Malignancies: A Clinicopathological Study. Ann. Surg..

[53] Di
Mola F. F., Di Sebastiano P. (2008). Pain and Pain Generation in Pancreatic
Cancer. Langenbecks Arch. Surg..

[54] Bapat A. A., Hostetter G., Von Hoff D. D., Han H. (2011). Perineural Invasion
and Associated Pain in Pancreatic Cancer. Nat.
Rev. Cancer.

[55] Zhu Z., Friess H., Di Mola F. F., Zimmermann A., Graber H. U., Korc M., Büchler M. W. (1999). Nerve Growth
Factor Expression Correlates with Perineural Invasion and Pain in
Human Pancreatic Cancer. J. Clin. Oncol..

[56] Di
Martino F., Barca P., Barone S., Bortoli E., Borgheresi R., De Stefano S., Di Francesco M., Faillace L., Giuliano L., Grasso L., Linsalata S., Marfisi D., Migliorati M., Pacitti M., Palumbo L., Felici G. (2020). FLASH Radiotherapy with Electrons: Issues related to
the production, monitoring, and dosimetric characterization of the
beam. Front. Phys..

