# Supplementary material for: FLASH Radiotherapy Enhances the Therapeutic Ratio in an Embryonic In Vivo Model of Pancreatic Carcinoma
Source: ACS Appl Mater Interfaces. 2025 Nov 7;17(46):63286–95. doi: 10.1021/acsami.5c19638 (PMC12635963; doi:10.1021/acsami.5c19638)
Supplement: Supplementary file 1 [file am5c19638_si_001.pdf]

Supporting Information

FLASH Radiotherapy Enhances the Therapeutic Ratio in an Embryonic In Vivo Model of Pancreatic Carcinoma

Noemi Giannini[1,2,3,4,5], Alessandra Gonnelli[1,2,4,5], Giovanni Gadducci[1,2,3,4,5], Paola Puccini[1,2], Andrea Cavalieri[4,5], Luigi Masturzo[4], Jake Harold Pensavalle[4,6], Mariagrazia Celentano[4], Fabio Di Martino[4,5,7], Federico Di Cocco[8], Cristian Scatena[8,9], Antonio Giuseppe Naccarato[8,9], Michele Menicagli[10], Patrizia Sarogni[1], Valentina Frusca[1,11], Dania Cioni[12], Giovanni Donato Aquaro[*,12], Valerio Voliani[*,§,1,13,14], Fabiola Paiar[*,§,2,3,4,5]

1 Center for Nanotechnology Innovation@NEST– Istituto Italiano di Tecnologia, Piazza San Silvestro 12, 56127 Pisa, Italy

2 Radiation Oncology Unit, Pisa University Hospital "Azienda Ospedaliero-Universitaria Pisana", Via Roma 67, Pisa 56126, Italy

3 Department of Translational Research and New Technologies in Medicine and Surgery, University of Pisa, Pisa, Italy

4 Centro Pisano Multidisciplinare Sulla Ricerca e Implementazione Clinica Della Flash Radiotherapy (CPFR), University of Pisa, Pisa, Italy

5 Center for Instrument Sharing of the University of Pisa (CISUP), University of Pisa, Italy

6 Sordina IORT Technologies S.p.A., Research and development, Aprilia, Italy

7 Unit of Medical Physics, Pisa University Hospital "Azienda Ospedaliero-Universitaria Pisana", via Roma 67, Pisa 56126, Italy

8 Division of Pathology, Department of Translational Research and New Technologies in Medicine and Surgery, University of Pisa, Pisa, Italy.

9 Department of Oncology, Pisa University Hospital, Pisa, Italy.

10 Fondazione Pisana per la Scienza ONLUS, via Ferruccio Giovannini 13, S. Giuliano Terme 56017, Pisa, Italy

11 Scuola Superiore Sant'Anna, Piazza Martiri della Libertà, 33, Pisa 56127, Italy

12 Unit of Radiology, Pisa University Hospital "Azienda Ospedaliero-Universitaria Pisana", via Roma 67, Pisa 56126, Italy

13 Department of Pharmacy, School of Medical and Pharmaceutical Sciences, University of Genoa, Viale Cembrano 4, Genoa 16148, Italy

14 Inter-University Center for the Promotion of the 3Rs Principles in Teaching & Research (Centro 3R), 56122 Pisa, Italy.

*Corresponding Authors: giovanni.aquaro@unipi.it, valerio.voliani@unige.it, fabiola.paiar@unipi.it.

[§]Shared senior authorship: valerio.voliani@unige.it, fabiola.paiar@unipi.it.

1. Supplementary Figures

Figure S1.

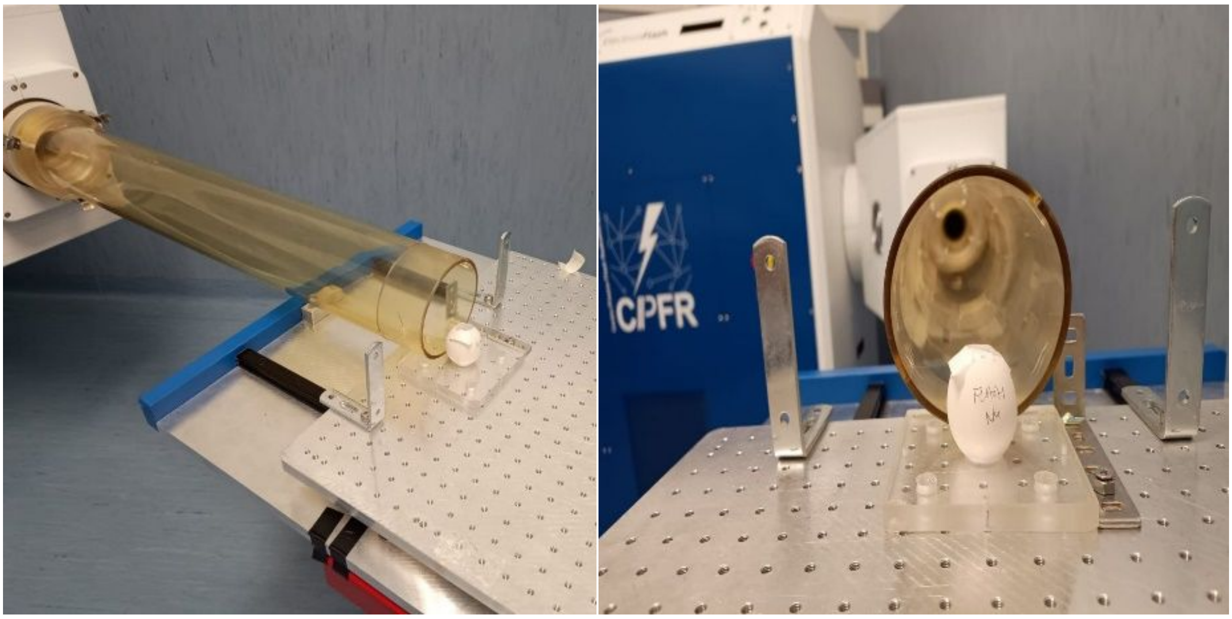

Figure S1. Experimental setup employed for CAMs irradiation.

Figure S2.

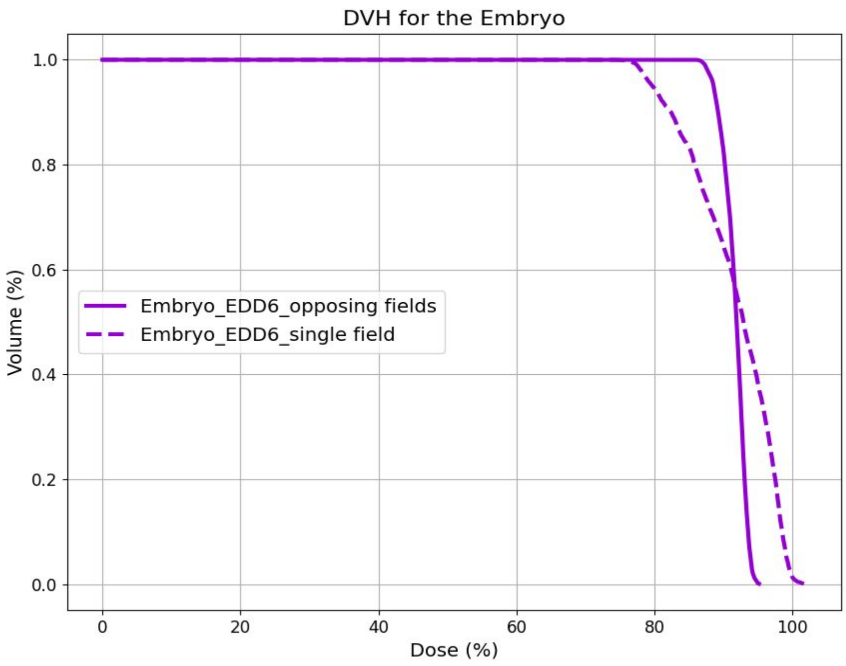

Figure S2. Distribution of radiation dose in embryos exposed to 8 Gy.

Figure S3.

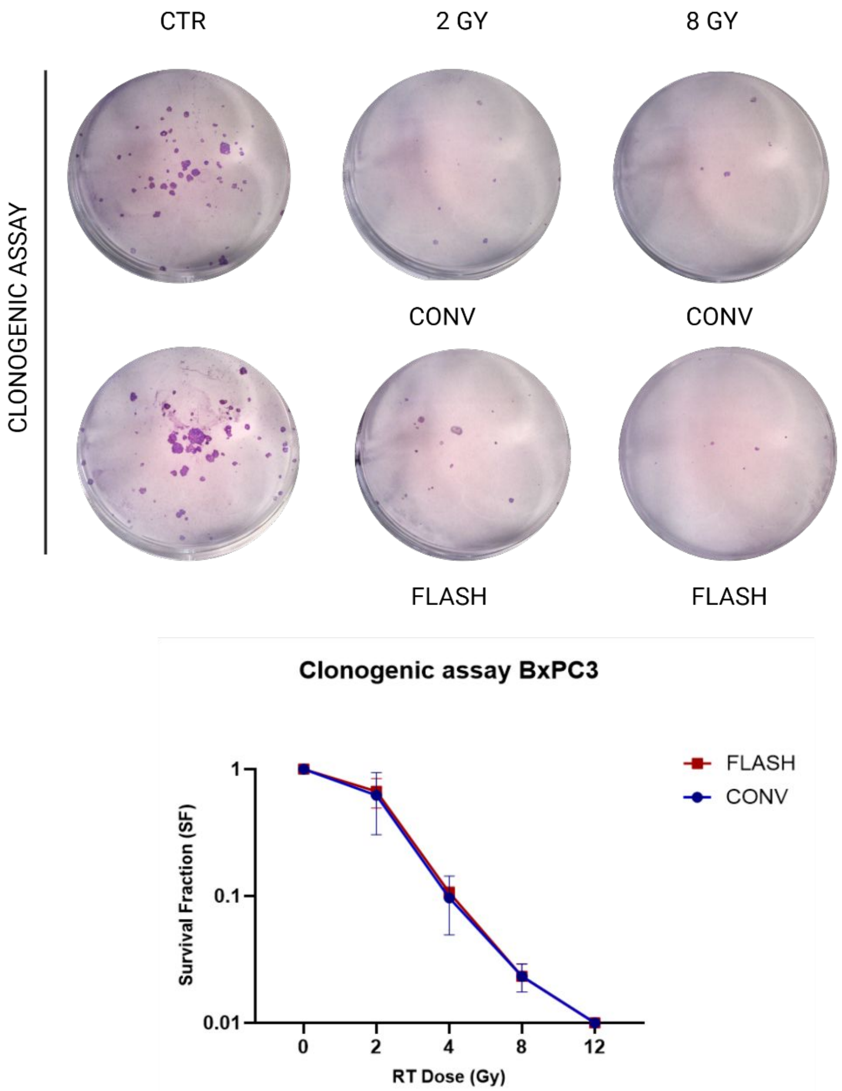

Figure S3. Clonogenic survival of BxPc3 pancreatic cancer cells following CONV and FLASH irradiation. Representative images of clonogenic assays (top) show colony formation under control conditions (CTR) and after exposure to 2 Gy and 8 Gy of CONV or FLASH radiotherapy. The quantitative analysis (bottom) presents the survival fraction (SF) as a function of radiation dose, highlighting a comparable dose–response relationship between CONV and FLASH modalities. Data are reported as mean ± SEM from three independent experiments.

Figure S4.

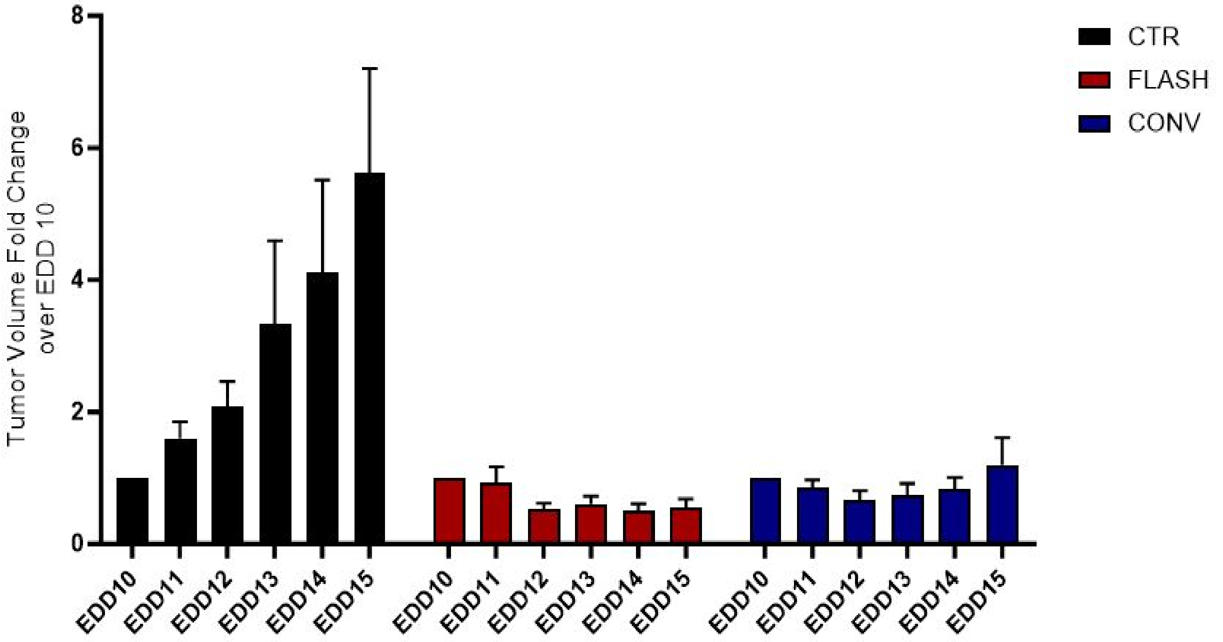

Figure S4. Tumor volume fold changes relative to EDD10 in: control (CTR), FLASH-irradiated (8 Gy), and conventionally irradiated (CONV 8 Gy) groups. In the CTR group, tumor volume increased progressively up to EDD15, with a marked fold change by EDD14–15. By contrast, both FLASH-RT and CONV-RT groups showed stable tumor volumes over the same timepoints, with minimal difference of fold change. These results indicate that both irradiation modalities effectively inhibited tumor progression. Data are reported as mean ± SEM of three independent experiments and at least 12 eggs per condition.

Figure S5.

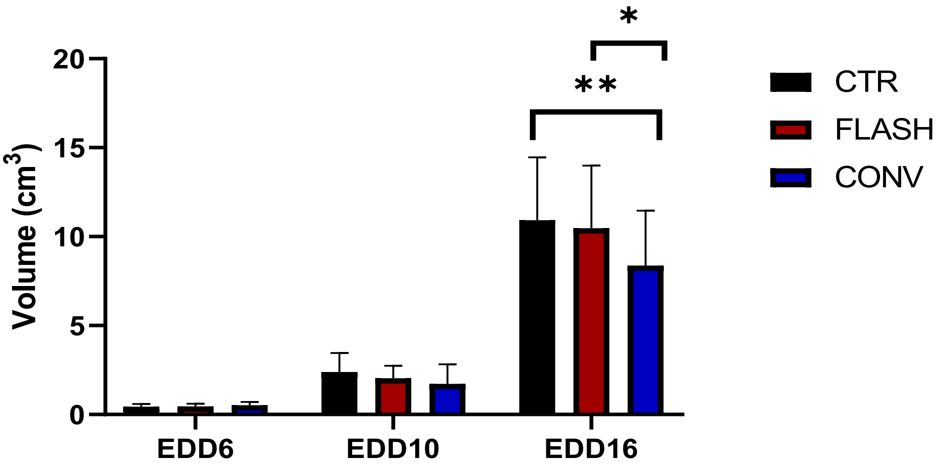

Figure S5. Embryo volume measurements at EDD6, 10, and 16 in control (CTR), FLASH-irradiated (8 Gy), and conventionally irradiated (8 Gy) groups. While all groups showed an increase in volume over time, a significant reduction in growth was observed in the CONV-RT group compared to CTR (p=0.0001), and a modest but significant difference was also noted between FLASH-RT and CONV-RT groups (p=0.0029) at EDD16. No significant differences were detected at EDD6 and EDD10, consistent with minimal variability at baseline. Data are reported as mean ± SEM of five independent experiments (100 eggs, 31 CTR group-35 FLASH group-34 CONV group).

Figure S6.

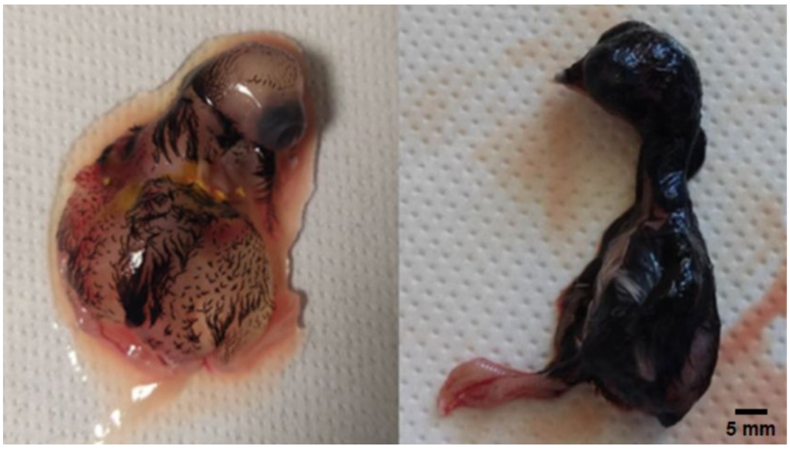

Figure S6. Common signs of embryo toxicity (such as alopecia and edema) caused by conventional radiotherapy.
